# Exploratory analysis of shoulder motion strategies for achieving functional hand behind the back in controls and after reverse shoulder arthroplasty. A study from the LaTour group

**DOI:** 10.1007/s00264-026-06987-w

**Published:** 2026-08-22

**Authors:** Gabriel Levy, Caecilia Charbonnier, Philippe Collin, Anthony Pernoud, Jean Gaillard, Alexandre Lädermann

**Affiliations:** 1 Division of Orthopaedics and Trauma Surgery, Hôpital de la Tour, Meyrin, Switzerland; 2 Medical Research Department, Artanim, Meyrin, Switzerland; 3 American Hospital of Paris, Neuilly-sur-Seine, France; 4 Orthopedics and Trauma Department, Centre Hospitalier Universitaire de Nantes, Nantes, France; 5 FORE (Foundation for Research and Teaching in Orthopedics, Sports Medicine, Trauma, and Imaging in the Musculoskeletal System), Meyrin, Switzerland

**Keywords:** Prosthesis, Complication, Internal rotation, PROMs, Outcomes, Results, Grammont

## Abstract

**Introduction:**

After reverse shoulder arthroplasty (RSA), most patients recover a satisfactory anterior forward elevation and external rotation elbow at side. However, the recovery of functional hand behind the back (fHBB), essential for daily life activities, remains unpredictable. This study aims to describe the complex mechanisms enabling fHBB and to explore their association with patient anatomy.

**Methods:**

A retrospective comparative study was conducted on patients who underwent RSA and a control group, all of whom had upper limb motion capture analysis. Baseline demographic and anatomical characteristics were collected for each subject. In the control group, humeral torsion, glenoid version, and scapular type were assessed through computer tomography (CT) imaging. For both the RSA and control groups, maximal glenohumeral range of motion (ROM)—flexion/extension, adduction/abduction, and internal/external rotations—were quantified using an optical motion capture system and 3D reconstructions. Movements used to reach the highest spinal level possible were then classified into distinct strategies using clustering. In controls, these movement strategies were correlated with anatomical parameters (humeral torsion, glenoid version, and scapular type). RSA patients were then descriptively projected onto the control clusters to explore similarities. Within the RSA cohort, ROM were compared between patients who succeeded or failed to achieve fHBB (hand above L5).

**Results:**

Among the controls who reached HBB type 3 (above T12) (*n *= 45 shoulders), an extension-dominant cluster (*n* = 25, extension 24° ± 9°, abduction 2° ± 5°, IR 27° ± 8°) and an internal rotation dominant group (*n* = 20, extension 17° ± 7°, abduction 5° ± 11°, IR 53° ± 10°) were identified. No statistically significant differences in humeral torsion (*p* = 0. 534), glenoid version (*p* = 0. 229), or scapular orientation (*p* = 0. 825) were found between the two clusters. In the RSA group, six out of ten reached fHBB. The successful and unsuccessful groups did not differ in terms of extension (17° ± 11° vs. 20° ± 12°, *p* = 0.761) or abduction (15° ± 12° vs. 8° ± 2°, *p* = 0.257). Taken together, these exploratory findings point to IR and extension as candidate contributors to fHBB that merit confirmation in larger cohorts.

**Conclusion:**

This exploratory study identified two strategies for achieving HBB above T12: extension-dominant and internal rotation-dominant, emphasizing the complex, multiplanar kinematics required to reach the back. Patients with RSA who regained fHBB (above L5) showed a tendency for increased IR but did not show significant differences from the unsuccessful patient suggesting that other factors are also involved.

**Supplementary Information:**

The online version contains supplementary material available at https://doi.org/10.1007/s00264-026-06987-w.

## Introduction

After reverse shoulder arthroplasty (RSA), a majority of patients recover satisfactory anterior forward elevation and external rotation [5, 28]. However, recovery of functional hand behind the back above L5 (fHBB), remains unpredictable [10]. fHBB is, however, an essential motion to perform activities of daily living such as perineal hygiene, unclasping a bra, threading a belt, or tucking in a shirt. The unpredictability of return of fHBB in one or both shoulders may even deter some surgeons from performing bilateral RSA [33].

Restoration of the fHBB requires a multiplanar complex movement involving probably extension, abduction, and internal rotation (IR). Moreover, several anatomical and muscular factors about the shoulder are implicated in fHBB recovery, such as subscapularis tendon repair [11, 13, 24] and/or healing, [10] prosthetic designs and demographics [20–22, 26, 32]. Among the latter, it has been shown that postoperative fHBB restoration is associated with preoperative range of motion (ROM), [19] body mass index (BMI), [12, 26] smoking status, male gender, and American Society of Anesthesiologists (ASA) score [16]. These findings may explain why planning softwares do not seem to properly anticipate HBB; a study showed that preoperative Blueprint (Stryker, Bloomington, MN, USA) 3D CT planning software is unable to accurately predict ROM one year after RSA [3].

This exploratory study aimed to describe shoulder movement strategies associated with fHBB in healthy controls, and to characterize the ROM in RSA patients. The hypothesis was that some combination of movements in different planes would allow for better overall HBB.

## Materials and methods

### Study design, data collection, and ethical committee approval

A retrospective analysis of patients who had undergone motion capture analysis of the upper limbs was performed. All patients had been included in various research protocols approved by the local ethical committee (CCER 2019–02469 trial registration number NCT04479397, AMG, 18–12), Shoulder OUTcome (SHOUT) multicenter study (CCER 14–277) and provided written informed consent for their participation and the use of their data and images for research and publishing purposes. Inclusion criteria were: (1) patients who had undergone motion capture of the upper limbs with HBB determination with or without RSA. The exclusion criteria were as follows: 1) incomplete ROM analysis, 2) contra-indication to magnetic resonance imaging (MRI), magnetic resonance arthrography (MRA) or computer tomography (CT) scan.

### Study variables

The outcomes of interest were the glenohumeral ROM in all planes ─ specifically extension, abduction/adduction, and IR ─ to reach the highest spinal level in a population with and without RSA. Control cases were assessed for humeral torsion, glenoid version, scapular internal rotation and scapular type according to Moroder [25]. The following baseline characteristics were assessed: age, sex, and shoulder side.

### Equity, diversity and inclusion statement

The studied population included women and men from various countries in Southern Africa, North America, Oceania, and Europe, reflecting the usual distribution for this pathology. Additionally, the cohort represented a wide range of ages, backgrounds, and demographics.

The research team comprised one woman and four men from Central Europe at different stages of their careers and clinical disciplines. No data on race, socioeconomic status, or other social determinants were collected.

### Radiological assessment and motion capture analysis

All volunteers underwent an MR shoulder arthrography or a CT scan. The MRI examinations were conducted after a fluoroscopically guided arthrography with a contrast agent and with an anterior approach. MRI was performed with a 1.5 T HDxT system (General Electric Healthcare, Milwaukee WI, USA). A dedicated shoulder surface coil was used. A sagittal T1 weighted fast spin echo sequence, a coronal and sagittal T2 weighted fast spin echo sequence with fat saturation, a coronal and axial T1 weighted fast spin echo sequence with fat saturation, and three 3D fast gradient echo (Cosmic® and Lava®) sequences were achieved. The CT examinations were conducted with a LightSpeed VCT 64 rows system (General Electric Healthcare, Milwaukee, WI). Images were acquired at 0.63 mm slice thickness. Based on the 3D MR or CT images, patient-specific 3D models of the shoulder bones (humerus, scapula, clavicle and sternum) were reconstructed for each volunteer using Mimics software (Materialize NV, Leuven, Belgium).

In controls, the anatomic measurements were made on CT-scan with axial views. Humeral torsion was measured as the angle between the interepicondylar line and the perpendicular line to the articular surface of the humeral head. The glenoid version was measured as the angle between the line perpendicular to the axis of the scapula, and the glenoid surface. Scapular types (A, B, or C) were determined based on the angle between the axis of the scapula and the perpendicular to the sagittal plane measured as the line bisecting the vertebrae [25].

**Fig. 1:**
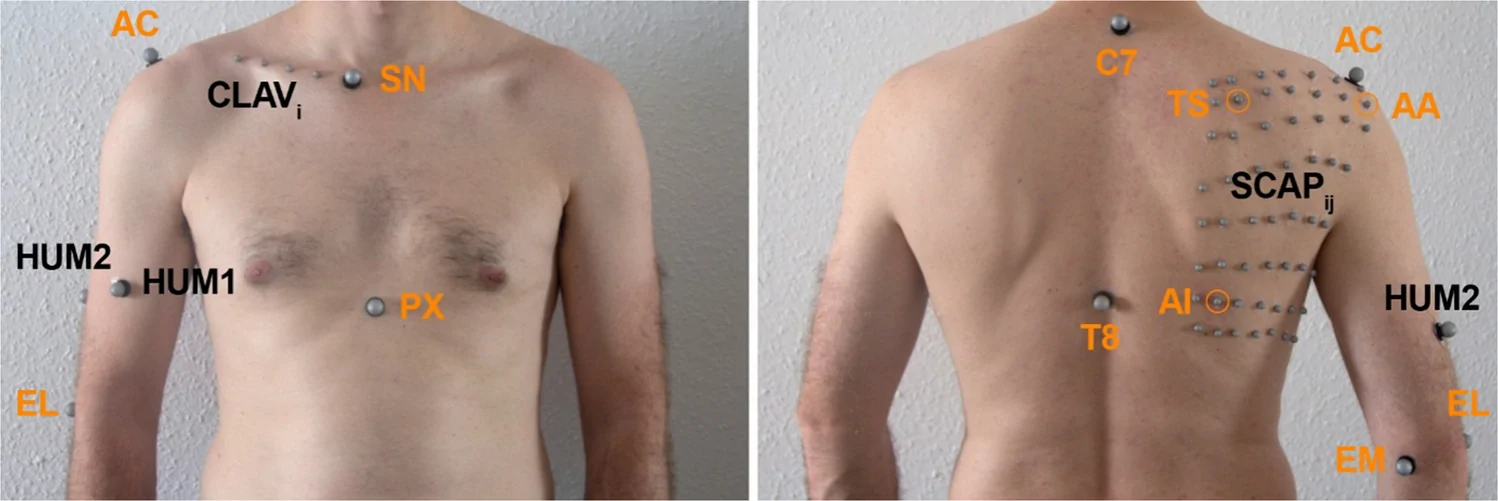
Marker placement, including markers placed on anatomical landmarks (orange) and technical markers (black). PX = xyphoid process, SN = sternal notch, AC = acromion, TS = trigonum spinae, AA = angulus acromialis, AI = angulus inferior, EL = lateral epicondyle, EM = medial epicondyle

Kinematic data were recorded using a Vicon MX T-Series motion capture system (Vicon, Oxford Metrics, UK) consisting of 24 cameras (24 × T40S) sampling at 120 Hz. The subjects were equipped with spherical retroreflective markers placed directly onto the skin using double-sided adhesive tape (Fig. 1). Four markers (Ø 14 mm) were attached to the thorax (sternal notch, xyphoid process, C7 and T8 vertebra). Four markers (Ø 6.5 mm) were placed on the clavicle. Four markers (Ø 14 mm) were fixed on the upper arm, two placed on anatomical landmarks (lateral and medial epicondyles) and two as far as possible from the deltoid. For the scapula, one marker (Ø 14 mm) was fixed on the acromion. In addition, the scapula was covered with 56 markers (Ø 6.5 mm) to form a 7 × 8 regular grid. Finally, additional markers were distributed over the body (non- dominant arm and legs) for visualization purposes only.

After appropriate warm-up, participants were asked to perform the following movements: 1) forward elevation from 0° to 180°, 2) abduction from 0° to 180° in the scapular plane, 3) IR/ER elbow at the side, and 4) HBB. Three trials were recorded. The same investigators attached all markers and performed all measurements.

**Fig. 2:**
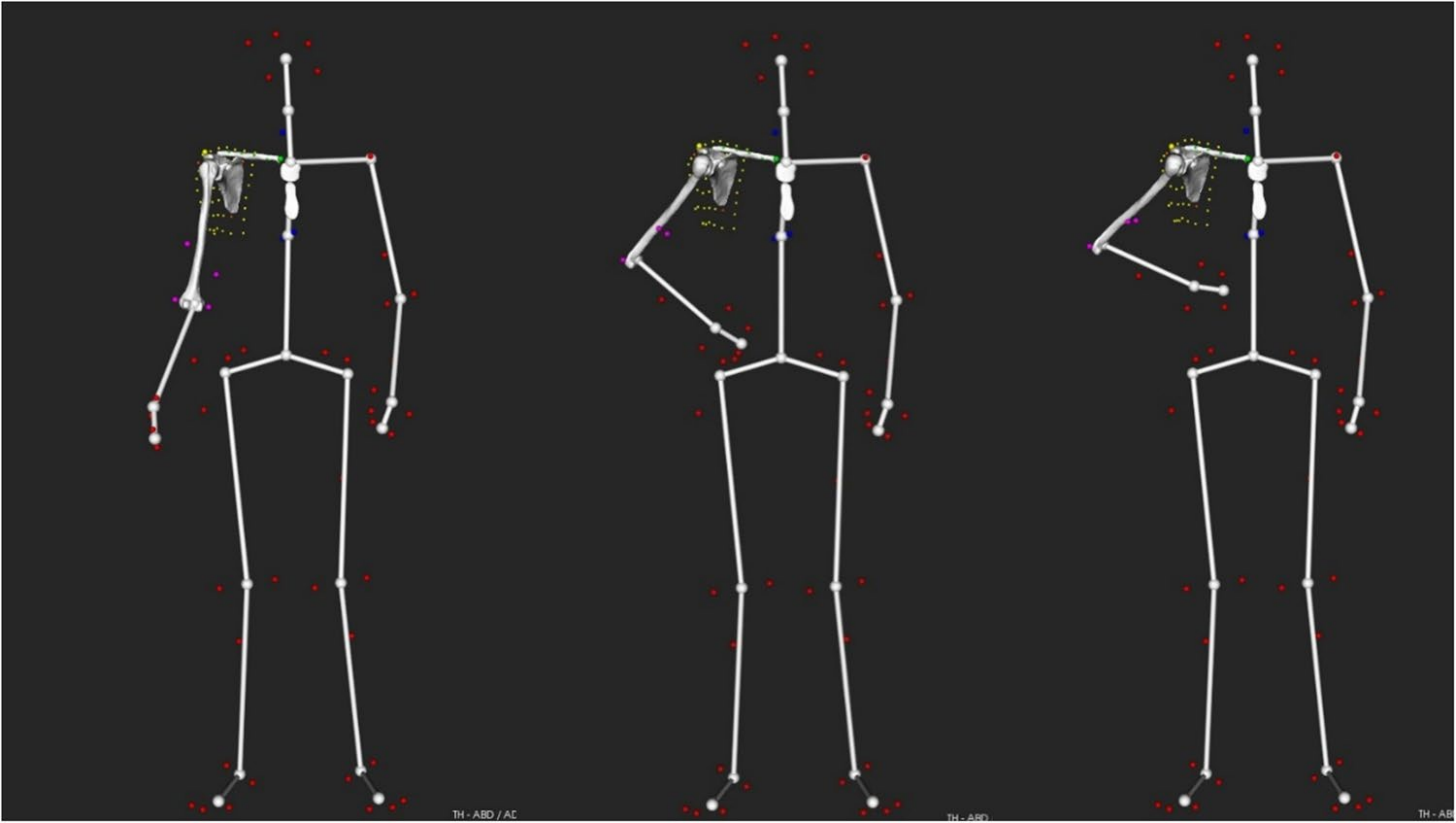
Kinematic animation of the shoulder (here the right shoulder joints) during IR1. Note: A ball and stick representation of the overall skeleton was also added to improve the analysis and visualization of the motion. Reprinted with permission from www.BeeMed.com

Shoulder kinematics was computed with custom-made software from the recorded markers’ trajectories using a validated biomechanical model which accounted for skin motion artifact [6, 7]. The model was based on a patient-specific kinematic chain using the shoulder 3D models reconstructed from the MRI or CT data and a multi-body optimization algorithm with loose constraints on joint translations (accuracy: translational error < 3 mm, rotational error < 4 degrees). As a result, the computed motions were applied to the shoulder 3D models reconstructed from their MRI and CT data (Fig. 2).

**Fig. 3:**
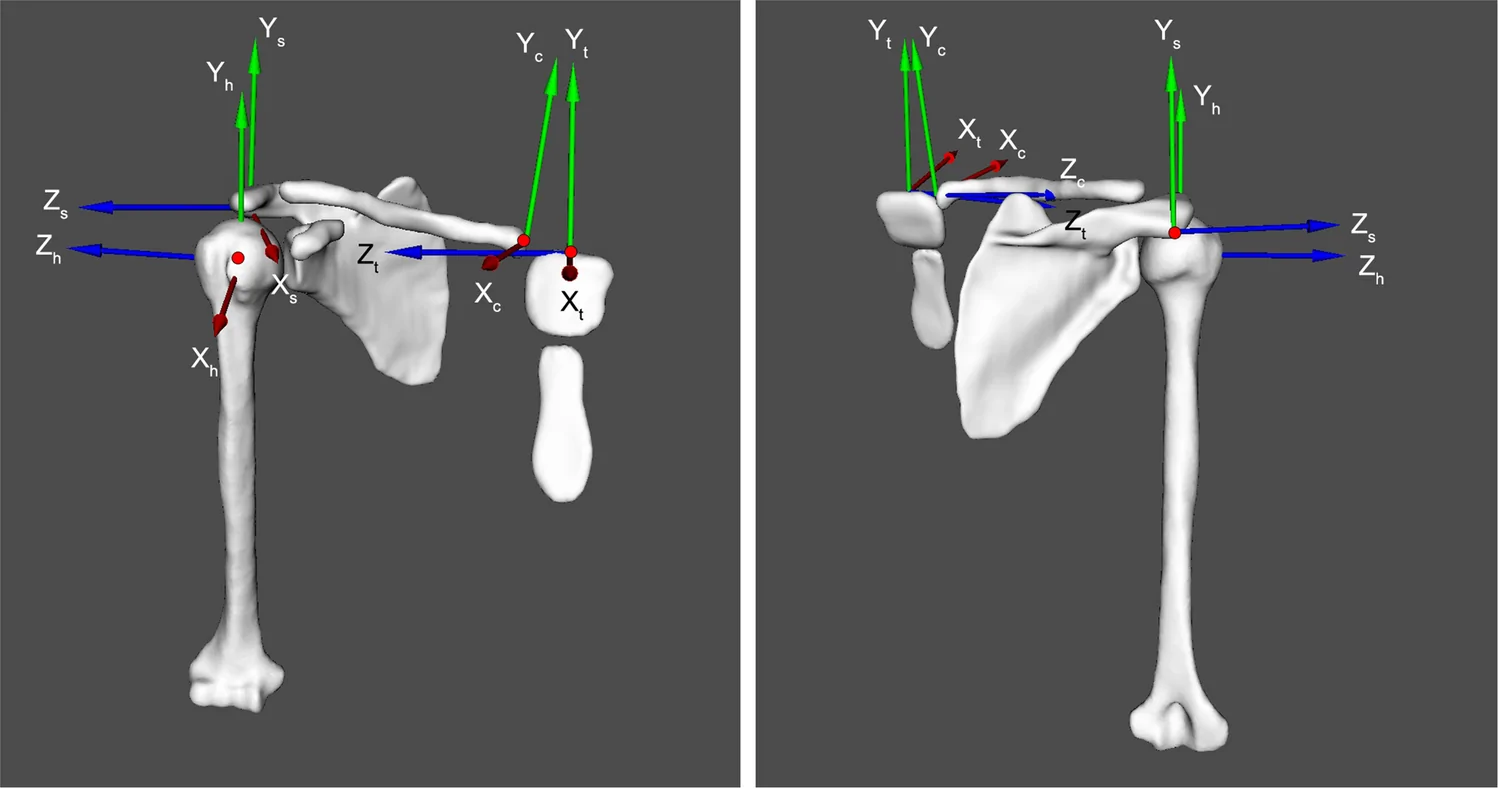
Bone coordinate systems for the thorax (Xt Yt Zt), clavicle (Xc Yc Zc), scapula (Xs Ys Zs) and humerus (Xh Yh Zh) Reproduced from Charbonnier et al. [6] with permission

To permit ROM description of the shoulder kinematic chain, local coordinate systems (Fig. 3) were established based on the definitions suggested by the International Society of Biomechanics [34] to represent the thorax, clavicle, scapula and humerus segments using anatomical landmarks identified on the subject’s bony 3D models. The glenohumeral joint center was calculated based on a sphere fitting method [29] that fits the optimal sphere to the humeral head using the points of the 3D humeral model.

ROM were quantified for each movement at critical position and the angles recorded. This was obtained by calculating the relative orientation between the scapula and humerus coordinate systems at each point of movement and then expressed in clinically recognizable terms (flexion/extension, adduction/abduction and internal/external rotation) by decomposing the relative orientation into three successive rotations. These computations were performed independently from the major anatomical planes (i.e., sagittal, transverse, frontal planes).

The functional classification described by Collin et al. [10] was used to assess HBB:

- Type I: Unable to perform ─ the patient could not reach their ipsilateral hand behind their back (buttock/sacrum).
- Type II: The patient could reach their hand behind their back to at least waist level but required assistance by rolling, sliding, or walking their hands up their back/backsides (L5-L3).
- Type III: The patient could reach their hand behind their back to at least waist level in a fluid, uninterrupted fashion (T12 or more).

Type I was categorized as non-fHBB, while types II and III were considered as fHBB.

### RSA operative details

The RSA cohort comprised seven patients (three with primary glenohumeral osteoarthritis and four with irreparable rotator cuff tears) who underwent reverse shoulder arthroplasty with a lateralized design, 145° neck-shaft angle and short stem. Pre-operative HBB reached sacrum for five patients and T12 for five patients. Mean time from surgery to motion capture analysis was 17 months (range, 7 to 48 months). Regarding subscapularis repair status, three patients had successful subscapularis repair, two had subscapularis spared, and two were not repaired.

### Statistical analyses

Continuous variables were summarized as means ± standard deviations (SD) and medians with interquartile ranges (IQR). Normality was assessed visually and with the Shapiro–Wilk test.

Clustering analysis was performed to identify movement strategies among controls who successfully reached HBB type 3 according to Collin [8]. Extension, abduction, and internal rotation ROM values were included in the clustering. The optimal number of clusters was determined visually using the elbow and silhouette technique. K-means clustering was then performed to derive the final clusters. For each RSA patient, the Euclidean distance of their ROM values to the centroids of the clusters derived from healthy controls was computed. Each patient was then assigned to the cluster with the smallest distance (“closest cluster”) for subsequent analyses. Wilcoxon rank sum tests were used for between-cluster comparisons. Comparisons of scapular types across movement strategies were performed using Chi-square test.

Within the RSA group, the Wilcoxon rank sum test was used to compare ROM values between patients who did and did not successfully perform fHBB (above L5). Because combining patient-specific 3D bone models with 24-camera optical motion capture is resource-intensive, this study was designed as an exploratory, proof-of-concept analysis. Consequently, no a priori sample size calculation was performed. A two-sided significance level of p < 0.05 was used and p-values were reported without correction for multiple testing, because all statistical comparisons were exploratory in nature and intended to identify kinematic trends rather than definitive causal relationships [4, 27]. All statistical analyses were performed using R software (version 4.1.3, R Foundation for Statistical Computing, Vienna, Austria).

## Results

A total of 38 patients and 55 shoulders were analyzed in this study. The control group (*n* = 28 patients) had a median age of 39 (26 to 43) years. The RSA group (*n* = 10 patients) had a median age of 75 (71 to 78) years, were almost exclusively females (*n* = 9, 90%), and were mostly operated on their dominant side (*n* = 6, 60%). ROM analysis in the RSA group was performed at a mean of 17 (range, 7 to 48) months post-surgery.

### Strategies among controls with fHBB

**Fig. 4:**
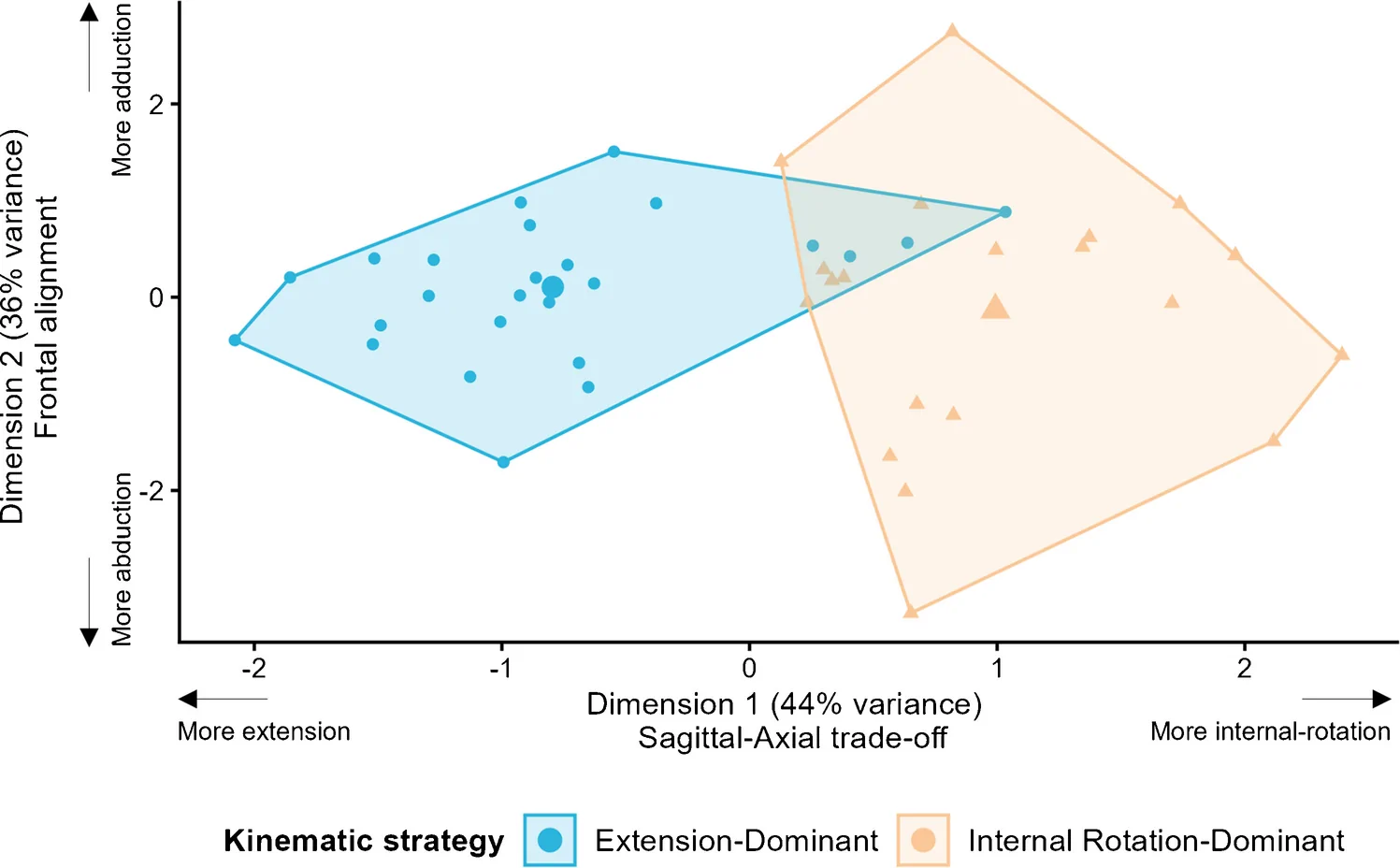
Principal Component Analysis (PCA) plot demonstrating the multiplanar kinematic clustering of functional hand-behind-the-back (fHBB) strategies in healthy controls (*n* = 45 shoulders). Two distinct kinematic clusters were identified: an Extension-Dominant strategy (Cluster A, blue) and an Internal Rotation-Dominant strategy (Cluster B, orange). Axis X (Dimension 1, 44% variance) primarily reflects a sagittal–axial trade-off between glenohumeral extension and internal rotation, while Axis Y (Dimension 2, 36% variance) is mainly associated with frontal-plane alignment through abduction/adduction. Each point represents one shoulder; shaded areas represent cluster dispersion (convex hulls). Large symbols indicate cluster centroids representing the mean position of each kinematic strategy in the PCA space

Among controls (28 patients, 45 shoulders), cluster analysis identified two distinct kinematic strategies to achieve HBB type 3: an extension-dominant strategy (group A) and an internal rotation-dominant strategy (group B) (Table 1, Fig. 4). Principal component analysis demonstrated that the first two dimensions accounted for 80% of the total kinematic variance. Dimension 1 (44%) primarily reflected a sagittal–axial trade-off opposing glenohumeral extension and internal rotation, whereas Dimension 2 (36%) was mainly associated with frontal plane adjustment through abduction/adduction (Fig. 4).

**Table 1:** Range of motion for the two strategies identified to reach functional hand behind back

	Group A (*n* = 25 shoulders) Extension-dominant	Group B (*n* = 20 shoulders) IR-dominant	*p*-value
Extension, °			
Mean ± SD	24 ± 9	17 ± 7	
Median (IQR)	26 (18 to 28)	18 (13 to 20)	0.004
Abduction*, °			
Mean ± SD	2 ± 5	5 ± 11	
Median (IQR)	2 (0 to 6)	2 (−1 to 14)	0.537
IR, °			
Mean ± SD	27 ± 8	53 ± 10	
Median (IQR)	28 (20 to 31)	51 (45 to 59)	< 0.001

*IQR* InterQuartile Range, *IR* Internal rotation, *SD* Standard Deviation

*Negative values indicate adduction

### Were there associations between anatomical characteristics and these strategies?

**Fig. 5:**
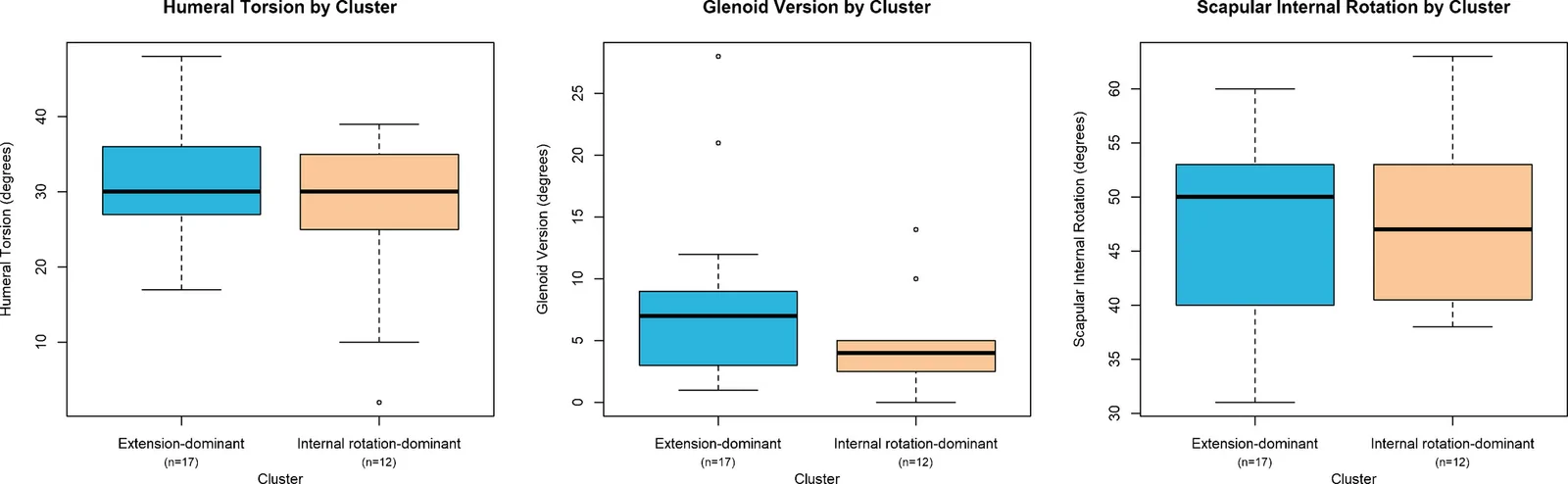
Distribution of humeral torsion, glenoid version, and scapular internal rotation amplitudes (degrees) across the two movement strategy-clusters in controls achieving hand behind back (HBB) type 3 (above T12). Each panel overlays all individual observations (jittered points) on the corresponding boxplot: the central bold line represents the median, the box edges the interquartile range (IQR, 25th–75th percentile), and the whiskers extend to the most extreme values within 1.5 × IQR. Sample sizes are indicated below each cluster, and the exact p-value of the between-cluster comparison (Wilcoxon rank-sum test) is shown on each panel

No statistical differences were observed across the two strategies for the humeral torsion (*p* = 0.534), glenoid version (*p* = 0.229), and scapular internal rotation (*p* = 0.825*)* (Table 2, Fig. 5). There was no association between scapular type and these strategies (*p* = 0.969).

**Table 2:** Anatomical characteristics between the different strategies identifed to reach functional hand behind back

	Group A (*n* = 17 shoulders) Extension-dominant	Group B (*n* = 12 shoulders) IR-dominant
Humeral torsion, °		
Mean ± SD	32 ± 8	27 ± 11
Median (IQR)	30 (27 to 36)	30 (25 to 35)
Glenoid version, °		
Mean ± SD	8 ± 7	5 ± 4
Median (IQR)	7 (3 to 9)	4 (3 to 5)
Scapular Internal rotation, °		
Mean ± SD	48 ± 9	48 ± 8
Median (IQR)	50 (40 to 53)	47 (41 to 53)

*IQR* InterQuartile Range, *IR* Internal rotation, *SD* Standard Deviation

### RSA group

In the RSA group, 6 out of 10 reached fHBB (above L5). Successful and unsuccessful patients did not differ significantly in glenohumeral extension (17° ± 11° vs. 20° ± 12°, *p* = 0.761) or abduction (15° ± 12° vs. 8° ± 2°, *p* = 0.257). Likewise, both groups did not differ statistically in glenohumeral IR, although successful patients showed numerically greater values (37° ± 20° vs. 17° ± 12°,* p* = 0.109), given the small sample this difference is reported as descriptive only. Out of the ten RSA patients, the four patients who failed to achieve fHBB were closest to the extension-dominant cluster. For the six successful RSA cases, four (67%) were closest to the extension-dominant group, and two (33%) to the internal-rotation dominant group. Taken together, these exploratory findings point to internal rotation and extension as candidate contributors to fHBB that merit confirmation in larger cohorts.

## Discussion

The ability to reach the back is crucial for daily tasks such as personal hygiene and dressing, and its absence can markedly reduce patient satisfaction. This study sets out to explore the complex mechanisms underlying the ability to perform HBB. In the control group, two distinct movement strategies were identified for performing HBB. Interestingly, patients with RSA who were able to reach the back did not favor one strategy over the other. This suggests that HBB is a complex movement, relying on both glenohumeral internal rotation and extension. Some level of abduction seems also necessary. The results of this study are consistent with the hypothesis that certain combinations of glenohumeral extension, abduction, and IR may facilitate HBB after RSA, although this remains exploratory given the small sample size.

### Primary outcome

Given the complexity of shoulder movements, careful preoperative planning has become essential to optimize postoperative mobility. While advances in implant design and surgical technique have improved ROM in several planes, HBB remains unpredictable [14, 17–19, 26, 30, 31]. Detailed ROM analysis in the present study suggested that individuals adopt different strategies to reach their back, with substantial variation across movement axes. After RSA, HBB tended to be reduced. The number of patients using an extension strategy to reach L5 was identical in the unsuccessful group, suggesting that extension is a necessary but insufficient component for recovering HBB function. Patients who favored an internal rotation strategy all reached L5, and the successful RSA tended to have greater IR. Also, successful patients showed greater glenohumeral abduction than controls with a balance between extension and internal rotation.

These findings suggest that surgical planning prioritizing extension while preserving internal rotation and abduction could improve functional outcomes. Supporting this concept, Collin et al. demonstrated the interdependence between internal rotation in abduction and fHBB [9]. While definitive clinical recommendations cannot be drawn from this exploratory work, these findings tentatively suggest that rehabilitation protocols targeting glenohumeral internal rotation and extension recovery may be of particular relevance after RSA. From a surgical perspective, implant configurations and glenosphere positioning strategies known to promote IR and extension clearance, as described in the existing literature [2, 15, 18, 35], may deserve further prospective evaluation in the context of fHBB restoration.

### Secondary outcomes

In the present cohort, humeral torsion and glenoid version for the control cases were comparable between the two movement strategies. While strictly exploratory, this preliminary finding tentatively suggests that the adoption of a specific fHBB strategy may not be exclusively dictated by static osseous alignment. Other unmeasured parameters, such as soft-tissue compliance and tensioning, muscle performance, motor control, and scapulothoracic behaviour, could potentially contribute to the observed postoperative performance. This is consistent with prior modeling work showing that implant configuration can substantially modify muscle–tendon length relationships and thereby influence soft-tissue tensioning in RSA [23].

When placed in the specific context of HBB after RSA, these results also support the notion that changing a single geometric parameter in isolation is unlikely to reliably restore fHBB. In clinical data, increasing glenosphere diameter does not appear to translate into better HBB: in a retrospective cohort comparing 36-mm vs. 39-mm glenospheres, HBB categories were similar and multivariable analysis did not identify glenosphere size as a determinant of fHBB [1].

In contrast, glenosphere positioning may be more relevant for the extension/abduction clearance required to HBB. In a virtual RSA model using a 135 degrees inlay humeral component, inferior placement (5 mm) increased extension and improved HBB, whereas posterior or posteroinferior eccentricity primarily increased extension and external rotation but tended to reduce isolated IR, suggesting a clearance-driven rather than a pure rotation-driven mechanism for HBB gains [2, 15, 18, 35].

Beyond implant position and bony impingement, scapulothoracic orientation likely remains a key, under-modeled determinant of HBB recovery. Moroder et al. demonstrated that scapular orientation (particularly IR and anterior tilt) is highly variable between individuals and closely related to thoracic posture [25]. The results of the present study are consistent with the broader concept that scapular contribution and posture-related orientation may influence HBB. Collin et al. also reported an association between increased scapular tilt and improved HBB after RSA [9]. This underlines the need to incorporate scapulothoracic orientation into preoperative planning and postoperative interpretation of HBB outcomes.

Finally, patient-related factors may modulate the observable HBB endpoint, even if they are not surgically modifiable. Smoking and higher ASA score have been associated with poorer HBB after RSA [16]. BMI has been inversely correlated with HBB-related activities of daily living [12].

### Limitations

This study has several limitations. First, the sample size, particularly in the RSA group, was relatively small, primarily due to the extensive time and resources required for subject recruitment, equipment setup, motion capture, and subsequent data processing. Because no a priori sample size calculation was performed, the RSA cohort analysis is admittedly underpowered to demonstrate definitive statistical significance. However, given that fHBB remains highly unpredictable after RSA, it is believed these preliminary data provide multiplanar kinematic trends that may inform future large-scale prospective trials. In addition, because this exploratory analysis involved multiple between-group comparisons without correction for multiplicity, the family-wise probability of a false-positive finding is increased; individual p-values should therefore be interpreted as hypothesis-generating rather than confirmatory, and require validation in adequately powered cohorts. Second, the analysis was limited to glenohumeral joint movements, without assessment of the scapulothoracic joint. Since the scapulothoracic joint also contributes to overall shoulder mobility, its exclusion represents a limitation that should be addressed in future research.

Finally, the focus of the analysis was on the final position achieved by the patient during motion capture. Although this approach allowed for the evaluation of different strategies for reaching the back, it did not account for the dynamics of the entire movement. A comprehensive analysis of the full movement trajectory may reveal additional factors influencing shoulder function and provide deeper insights into the underlying mechanisms.

## Conclusion

This exploratory study identified two strategies for achieving HBB above T12: extension-dominant and internal rotation-dominant, emphasizing the complex, multiplanar kinematics required to reach the back. Patients with RSA who regained fHBB (above L5) showed a tendency for increased internal rotation but did not show significant differences from the unsuccessful patient suggesting that other factors are also involved.

## Supplementary Information

Below is the link to the electronic supplementary material.

ESM 1 (DOC 81.5 KB)

## Data Availability

No datasets were generated or analysed during the current study.
