# Supplementary material for: Exploratory analysis of shoulder motion strategies for achieving functional hand behind the back in controls and after reverse shoulder arthroplasty. A study from the LaTour group
Source: Int Orthop. 2026 Aug 22;50(9):2385–94. doi: 10.1007/s00264-026-06987-w (PMC13562314; doi:10.1007/s00264-026-06987-w)
Supplement: Supplementary file 1 [file 264_2026_6987_MOESM1_ESM.doc]

STROBE Statement—Checklist of items that should be included in reports of ***case-control studies***

|  | Item No | Recommendation |
| --- | --- | --- |
| **Title and abstract** | 1 | (*a*) Indicate the study’s design with a commonly used term in the title or the abstract line 1 **Exploratory analysis** |
|  |  | (*b*) Provide in the abstract an informative and balanced summary of what was done and what was found. Done lines 9-46 |
| Introduction | | |
| Background/rationale | 2 | Explain the scientific background and rationale for the investigation being reported lines 54-70 |
| Objectives | 3 | State specific objectives, including any prespecified hypotheses lines 72-74 |
| Methods | | |
| Study design | 4 | Present key elements of study design early in the paper line 77 |
| Setting | 5 | Describe the setting, locations, and relevant dates, including periods of recruitment, exposure, follow-up, and data collection line 77-78 |
| Participants | 6 | (*a*) Give the eligibility criteria, and the sources and methods of case ascertainment and control selection. Give the rationale for the choice of cases and controls lines 77-93 |
|  |  | (*b*) For matched studies, give matching criteria and the number of controls per case |
| Variables | 7 | Clearly define all outcomes, exposures, predictors, potential confounders, and effect modifiers. Give diagnostic criteria, if applicable lines 89-93 |
| Data sources/ measurement | 8* | For each variable of interest, give sources of data and details of methods of assessment (measurement). Describe comparability of assessment methods if there is more than one group lines 104-181 |
| Bias | 9 | Describe any efforts to address potential sources of bias NA |
| Study size | 10 | Explain how the study size was arrived at lines 198-201 |
| Quantitative variables | 11 | Explain how quantitative variables were handled in the analyses. If applicable, describe which groupings were chosen and why lines 104-181 |
| Statistical methods | 12 | (*a*) Describe all statistical methods, including those used to control for confounding lines 184-205 |
|  |  | (*b*) Describe any methods used to examine subgroups and interactions lines 184-205 |
|  |  | (*c*) Explain how missing data were addressed lines 184-205 |
|  |  | (*d*) If applicable, explain how matching of cases and controls was addressed lines 184-205 |
|  |  | (*e*) Describe any sensitivity analyses lines 184-205 |
| Results | | |
| Participants | 13* | (a) Report numbers of individuals at each stage of study—eg numbers potentially eligible, examined for eligibility, confirmed eligible, included in the study, completing follow-up, and analysed lines 207-238 |
|  |  | (b) Give reasons for non-participation at each stage NA |
|  |  | (c) Consider use of a flow diagram NA |
| Descriptive data | 14* | (a) Give characteristics of study participants (eg demographic, clinical, social) and information on exposures and potential confounders lines 207-238 |
|  |  | (b) Indicate number of participants with missing data for each variable of interest lines 207-238 |
| Outcome data | 15* | Report numbers in each exposure category, or summary measures of exposure lines 207-238 |
| Main results | 16 | (*a*) Give unadjusted estimates and, if applicable, confounder-adjusted estimates and their precision (eg, 95% confidence interval). Make clear which confounders were adjusted for and why they were included lines 207-238 |
|  |  | (*b*) Report category boundaries when continuous variables were categorized lines 207-238 |
|  |  | (*c*) If relevant, consider translating estimates of relative risk into absolute risk for a meaningful time period lines 207-238 |

| Other analyses | 17 | Report other analyses done—eg analyses of subgroups and interactions, and sensitivity analyses |
| --- | --- | --- |
| Discussion | | |
| Key results | 18 | Summarise key results with reference to study objectives lines 242-249 |
| Limitations | 19 | Discuss limitations of the study, taking into account sources of potential bias or imprecision. Discuss both direction and magnitude of any potential bias lines 302-317 |
| Interpretation | 20 | Give a cautious overall interpretation of results considering objectives, limitations, multiplicity of analyses, results from similar studies, and other relevant evidence lines 242-249 |
| Generalisability | 21 | Discuss the generalisability (external validity) of the study results lines 242-249 |
| Other information | | |
| Funding | 22 | Give the source of funding and the role of the funders for the present study and, if applicable, for the original study on which the present article is based Lines 352-362 |

*Give information separately for cases and controls.

**Note:** An Explanation and Elaboration article discusses each checklist item and gives methodological background and published examples of transparent reporting. The STROBE checklist is best used in conjunction with this article (freely available on the Web sites of PLoS Medicine at http://www.plosmedicine.org/, Annals of Internal Medicine at http://www.annals.org/, and Epidemiology at http://www.epidem.com/). Information on the STROBE Initiative is available at http://www.strobe-statement.org.
